# Evaluation of vestibulo-ocular reflex with functional head impulse test in healthy individuals: normative values

**DOI:** 10.3389/fneur.2023.1300651

**Published:** 2023-11-22

**Authors:** Deniz Uğur Cengiz, Hatice Seyra Erbek, Sanem Can Çolak, Büşra Kurtcu, Sümeyye Demirel Birişik, Ercan Karababa, Buşra Kuşman, Emre Akgün Özdemir, Mehmet Işık, İsmail Demir

**Affiliations:** ^1^Department of Audiology, Faculty of Health Sciences, Inonu University, Malatya, Türkiye; ^2^Department of Otolaryngology, Faculty of Medicine, Lokman Hekim University, Ankara, Türkiye; ^3^Department of Audiology, Institute of Health Sciences, Inonu University, Malatya, Türkiye; ^4^Department of Audiology, Faculty of Health Sciences, Bingöl University, Bingöl, Türkiye; ^5^Department of Audiology, Gülhane Faculty of Health Sciences, University of Health Sciences, Ankara, Türkiye; ^6^Department of Audiology, Turgut Özal Medicine Center, Inonu University, Malatya, Türkiye; ^7^Department of Audiology, Institute of Health Sciences, Istanbul Gelişim University, İstanbul, Türkiye

**Keywords:** functional head impulse test, vestibulo ocular reflex, vestibular system, normative values, visual acuity

## Abstract

**Objectives:**

This study aimed to determine the normative values of the functional head impulse test (fHIT) test in healthy young adults.

**Materials and methods:**

The study included 100 individuals, 58 females and 42 males, aged 20–25 years. Beon Solution Zero Branco (TV) fHIT test was applied to all participants. FHIT results were analyzed in terms of lateral-posterior–anterior semicircular canals (SSCs), gender, and right–left ear.

**Results:**

In the fHIT test, for the lateral SSC the mean percent correct answer (%CA) values for 4,000–6,000°/s^2^ and 1,000–7,000°/s^2^ were 88.52 ± 9.04 and 89.91 ± 6.95, respectively; for the posterior SSC, the mean %CA values for SSC 3000–6,000°/s^2^ and 1,000–7,000°/s^2^ were 90.63 ± 8.69 and 91.16 ± 7.42, respectively; for the anterior SSC, the mean %CA values for 3,000–6,000°/s^2^ and 1,000–7,000°/s^2^ were 91.21 ± 7.96 and 91.49 ± 7.13, respectively. There was no statistically significant difference between the right and left ear in %CA values at all accelerations in all SSCs (*p* > 0.05). There was a statistically significant difference between genders in the mean %CA values for 6,000–7,000°/s^2^, 4,000–6,000°/s^2^, and 1,000–7,000°/s^2^ in the lateral SSC and 3,000–6,000°/s^2^ in the anterior SSC (*p* < 0.05).

**Conclusion:**

The FHIT is a noninvasive test battery that functionally evaluates the VOR of the six semicircular canals. In clinical use, the determination of normative values at all accelerations (1,000–7,000 degrees/s^2^) constitutes an important database for future studies to distinguish pathologic results.

## Introduction

The visual system plays a key role in every moment of daily life. It is critical in coordinating movements and maintaining balance. The ability of the visual system to stabilize an image on the retina during head movements depends on the activity of the vestibular system. The activity of each of these two systems depends on the frequency of head oscillation. For example, at low frequencies (<0.1 Hz), the visual system is dominant; at medium frequencies, the vestibular and visual systems interact together to stabilize gaze; at high frequencies (1–5 Hz), only the vestibular system is involved (1). One of the most important functions of the vestibular system is to maintain visual acuity by stabilizing gaze (2). The vestibulo-ocular reflex (VOR) stabilizes gaze during high-frequency head movements by moving the eyes in the opposite direction of head movement to fix the image on the retina. Therefore, a decreased VOR function impairs gaze stabilization, leading to decreased visual acuity with head or body movement (3). Vertigo, dizziness, and decreased visual acuity following active head or body movement are associated with dysfunction of the vestibular system. A comprehensive assessment of vestibular function is essential to determine the cause of all these conditions (4). Testing the VOR is crucial to assess the function of the vestibular organs as there is a direct neural connection between the semicircular canals (SSCs) and the extraocular muscles (5).

The Head Impulse Test developed by Halmagyi and Curthoys is a method for the diagnosis of vestibular disorders, and more recently, the Video Head Impulse Test (vHIT), a computerized system that allows the calculation of VOR gain and visualization of covert saccades, has been developed (6, 7). The saccades and VOR gain seen in the Video Head Impulse Test provide information about the loss of the SSC functions. However, it does not provide information about the functional efficiency of SSC-evoking head movements; that is, whether gaze stabilization is sufficient for clear vision. On the other hand, in individuals with normal VOR gain, gaze stabilization may not be achieved during head movements due to inadequate visual processing and blurred vision may occur (8). In recent years, a new technique called the Functional Head Impulse Test (fHIT), has been developed to identify vestibular symptoms that cannot be detected by standard vestibular tests and to evaluate dynamic visual acuity during head movements (9). The fHIT, a new vestibular test, is based on the ability to recognize the orientation of a Landolt optotype C that briefly appears on a computer screen, while passive head stimuli are applied by the clinician at various accelerations (1,000–7,000°/s^2^). The fHIT, which assesses the ability to see and read clearly during head movement, is a functional measure of the VOR. The fHIT provides information about the functional performance of the rotational VOR by measuring the ability to read and maintain clear vision during passive head impacts (10–13). However, like the head impulse test, the fHIT test may have some limitations. As in the HIT test, in the fHIT test, the stimulus is provided by the clinician and the test results may vary from person to person (14). It is also reported that the orientation of the labyrinth varies from person to person, therefore the head movement applied by the clinician may cause stimulation of different channel systems in people (15). In short, as in the HIT and vHIT test, the results in the fHIT test may depend on both the clinician and the patient.

Although fHIT measurements have been evaluated in different pathologies in a limited number of studies in the literature (3, 9, 10, 16), there is no normalization study of the fHIT in healthy young individuals. In our study, we aimed to determine the normal values of the fHIT in healthy young adults and to use it as normative values in the vestibular evaluation of patients with balance disorders.

## Materials and methods

### Individuals

Individuals aged 20–25 years, who did not have communication disabilities, cognitive problems, pre-existing balance problems, hearing problems and visual loss, and who voluntarily agreed to participate in the study by signing the Informed Consent Form were included in the study. The sample of the study was determined as 100 individuals with an effect size of 0.332, a margin of error of 0.05, a confidence level of 0.95, and a population representativeness of 0.95 according to the calculation made by power analysis using the G*power 3.1 program. One hundred and twenty individuals were evaluated in the study. Nine individuals were excluded from the study because of visual problems, 3 because of diagnosed hearing loss, and 8 because they could not complete the test. As a result, the study was completed with 100 individuals who met the inclusion criteria.

### Data collection

The study was conducted at a university in eastern Türkiye between May 2022 and July 2022. Voluntary sampling method, one of the non-probability sampling methods, was used to determine the participants. Individuals participating in the study were administered the fHIT test.

#### Functional head impulse test

A Beon Solution Zero Branco (TV) fHIT system was used in the study. The participant was positioned 1.5 meters away from the computer screen. The test was explained by placing a headband with a gyroscope on it. The participant was given a mini keyboard and asked to select the direction of the C-shaped optotype on the keyboard, which blinked on the computer screen within 80 milliseconds and shrank according to the answers given. Static visual acuity was determined by the minimum optotype size that the participant could read. The test phase started by increasing the optotype size by 0.6 LogMAR. The patient was asked to select the direction of the rapidly blinking optotype on the computer screen using the keyboard by making head movements at acceleration values of 1,000, 2,000, 3,000, 4,000, 5,000, 6,000 and 7,000 °/s^2^ in the direction of the SSC to be stimulated. The tests were completed in the lateral, RALP (right anterior left posterior) and LARP (left anterior right posterior) planes and the percentage of correct answers (%CA) was calculated.

**Figure 1:**
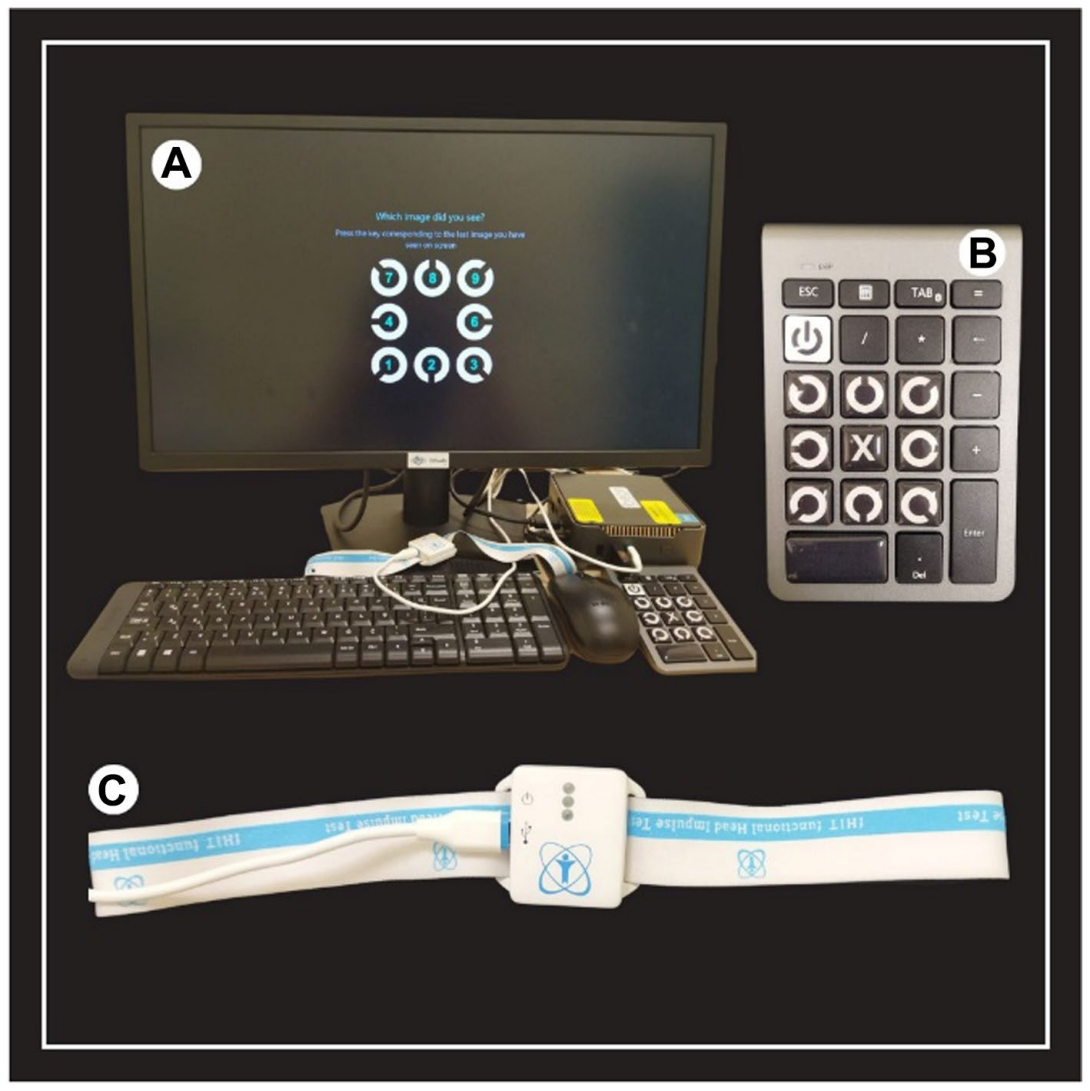
The set-up diagram of the f-HIT device. **(A)** Equipment of the f-HIT device. **(B)** Mini keyboard for determining the orientation of the Landolt C optotype in static and dynamic visual acuity. **(C)** Gyroscope to determine the velocity, acceleration and direction of the head.

The set up diagram of the f-HIT device is shown in Figure 1.

**Figure 2:**
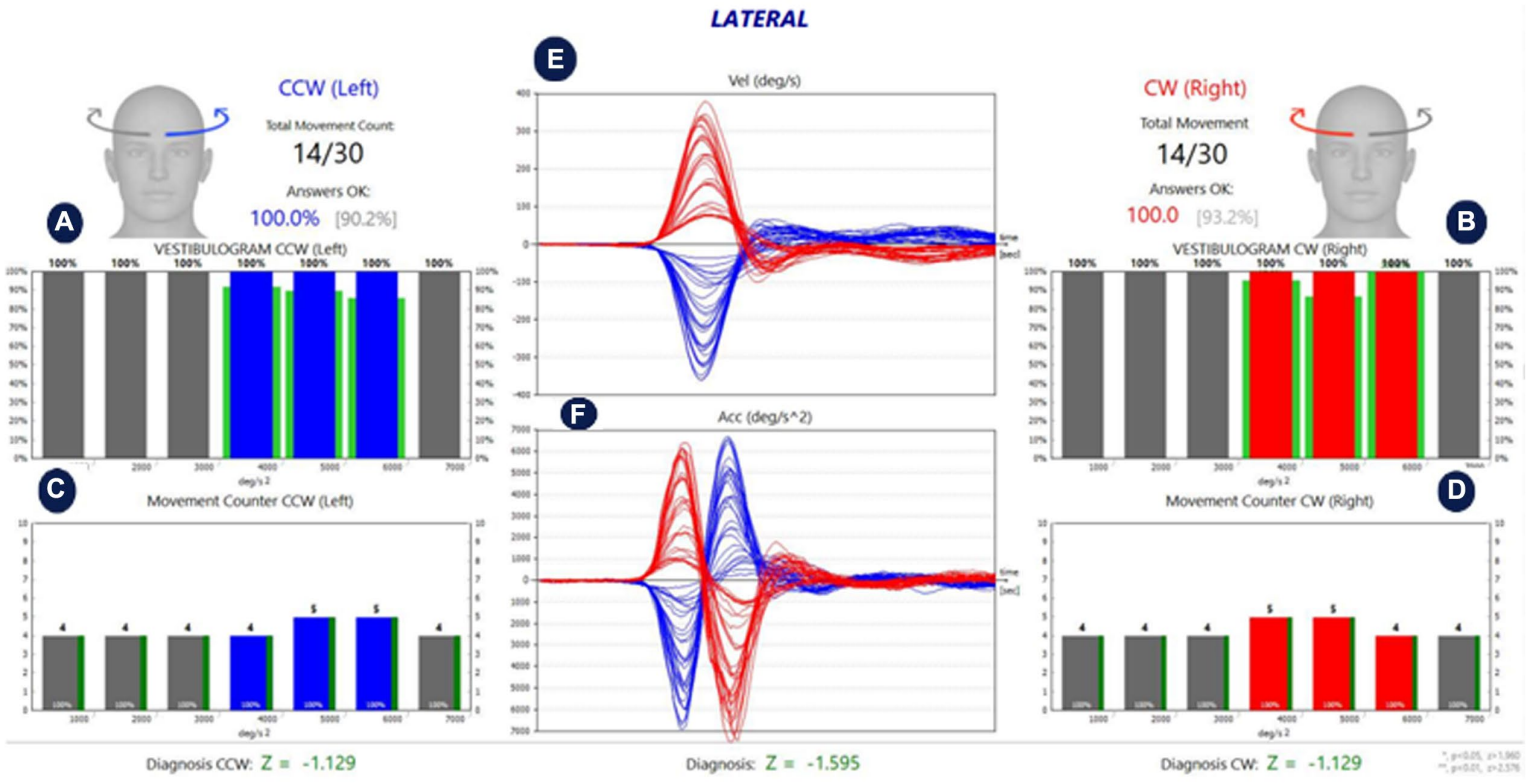
Analysis of lateral SCCs of a healthy individual. **(A,B)** Show the %CA value per 1,000 °/s^2^ acceleration values for left and right stimulation, respectively. **(C,D)** Show the number of head movements per 1,000 °/s^2^ acceleration values in left and right stimulation, respectively. While the green lines around the red and blue blocks in **(A,B)** show the normative values in the software, the green lines next to the red and blue blocks in **(C,D)** reflect the %CA value. The percentage value next to the head figure shows the average %*CA.* The value in parentheses indicates the normative value of the average %*CA.* **(E)** Is the velocity graph of head movement, and **(F)** is the acceleration graph. The Z parameter at the bottom of all tables is the statistical confidence degree index. If it exceeds the confidence threshold, it turns red, meaning that the difference between the subject’s results and the expected outcome for a healthy subject is sufficient to indicate a problem.

**Figure 3:**
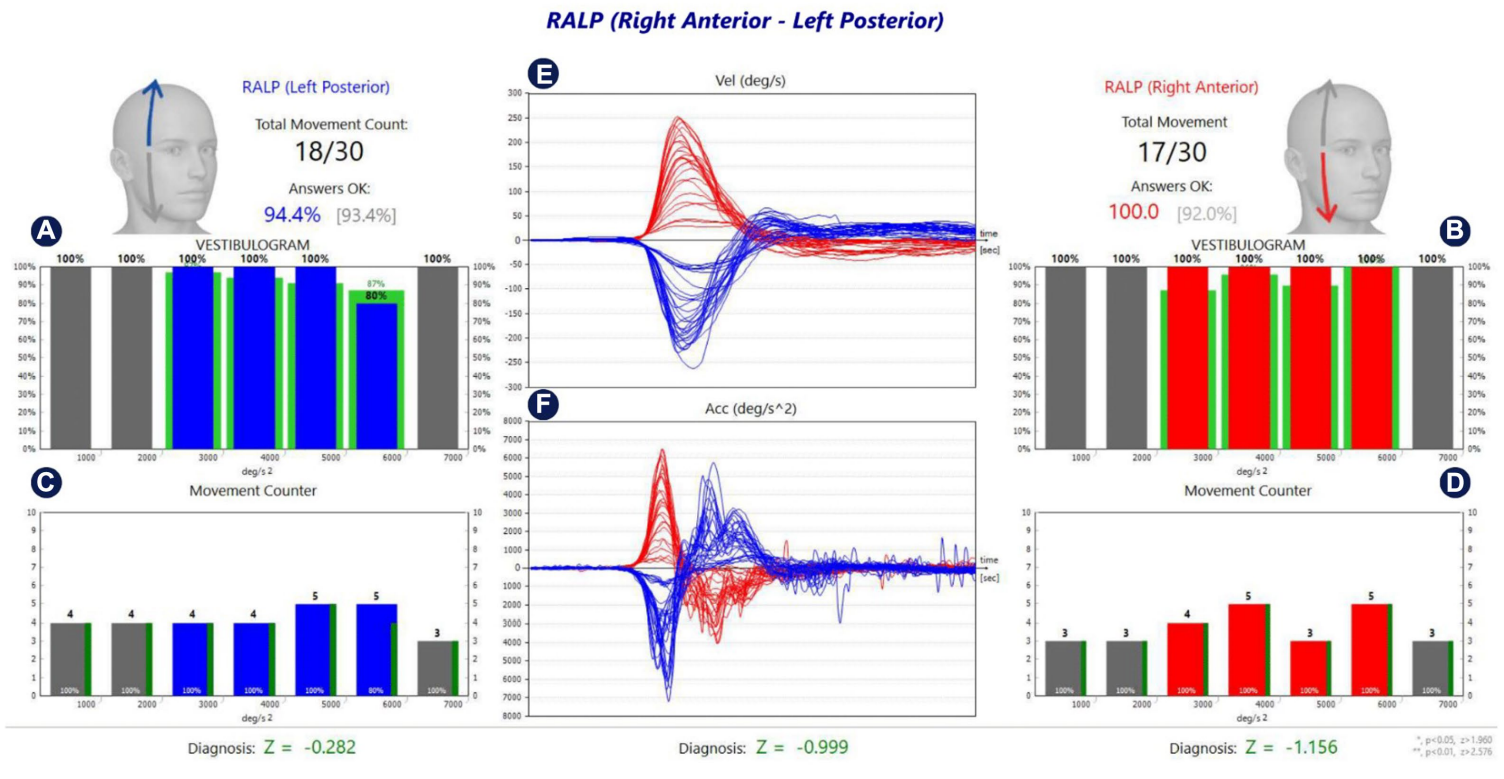
Analysis of RALP SCCs of a healthy individual. **(A,B)** Show the %CA value per 1,000 °/s^2^ acceleration values for left and right stimulation, respectively. **(C,D)** Show the number of head movements per 1,000 °/s^2^ acceleration values in left and right stimulation, respectively. While the green lines around the red and blue blocks in **(A,B)** show the normative values in the software, the green lines next to the red and blue blocks in **(C,D)** reflect the %CA value. The percentage value next to the head figure shows the average %*CA.* The value in parentheses indicates the normative value of the average %*CA.* **(E)** Is the velocity graph of head movement, and **(F)** is the acceleration graph. The Z parameter at the bottom of all tables is the statistical confidence degree index. If it exceeds the confidence threshold, it turns red, meaning that the difference between the subject’s results and the expected outcome for a healthy subject is sufficient to indicate a problem.

**Figure 4:**
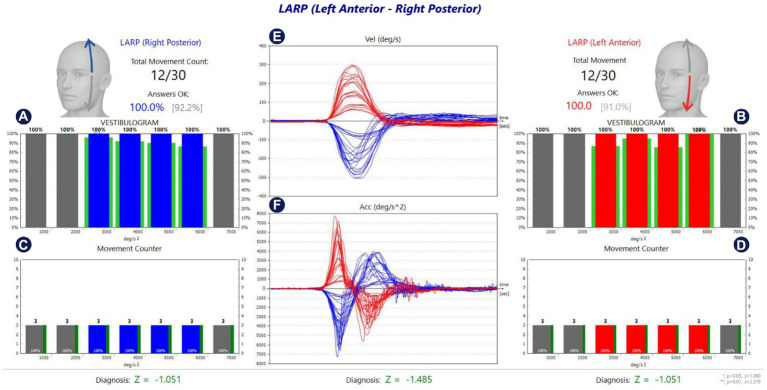
Analysis of LARP SCCs of a healthy individual. **(A,B)** Show the %CA value per 1,000 °/s^2^ acceleration values for left and right stimulation, respectively. **(C,D)** Show the number of head movements per 1,000 °/s^2^ acceleration values in left and right stimulation, respectively. While the green lines around the red and blue blocks in **(A,B)** show the normative values in the software, the green lines next to the red and blue blocks in **(C,D)** reflect the %CA value. The percentage value next to the head figure shows the average %*CA.* The value in parentheses indicates the normative value of the average %*CA.* **(E)** Is the velocity graph of head movement, and **(F)** is the acceleration graph. The Z parameter at the bottom of all tables is the statistical confidence degree index. If it exceeds the confidence threshold, it turns red, meaning that the difference between the subject’s results and the expected outcome for a healthy subject is sufficient to indicate a problem.

The analysis screen of the fHIT test performed in the lateral, RALP, and LARP SSC planes in a healthy individual is shown in Figures 2–4, respectively.

### Statistical analysis

The analysis of the data included in the study was performed with SPSS (Statistical Program in Social Sciences) 25 program. Whether the data included in the study conformed to the normal distribution was checked with the Kolmogorov Smirnov Test (17). The significance level (p) was set as 0.05 for comparison tests. Since the variables were not normally distributed (*p* > 0.05), the analysis was continued with nonparametric test methods. Comparisons in independent paired groups were performed with the Mann Whitney U test because the normality assumption was not met. In the analysis of categorical data, Chi-square (X^2^) analysis was performed by creating cross-tables. Spearman rank correlation coefficient was used in the relationships between numerical variables.

### Ethical principles of research

Approval was obtained from the University’s Institute of Health Sciences Non-Interventional Clinical Research Ethics Committee (decision number: 2022/3465) and all individuals participating in the study.

## Results

One hundred and twenty individuals were evaluated in the study. Nine individuals were excluded from the study because they had visual impairment, 3 were diagnosed with hearing loss, and 8 could not complete the test. As a result, the study was completed with 100 individuals who met the inclusion criteria. A total of 100 participants were included in the study, of which 58 (58%) were female and 42 (42%) were male. The age distribution of the participants varied between 20 and 25 years. While the mean age of the female participants was 21.00 ± 0.95, the mean age of the male participants was 21.33 ± 1.30. There was no statistically significant difference between both genders in age values (*p* > 0.05).

In the fHIT test applied, the mean %CA values for 4,000–6,000°/s^2^ and 1,000–7,000°/s^2^ were 88.52 ± 9.04 and 89.91 ± 6.95, respectively, as a result of the stimulation in the lateral SSC direction. The mean %CA values of 3,000–6,000°/s^2^ and 1,000–7,000°/s^2^ were 90.63 ± 8.69 and 91.16 ± 7.42, respectively, as a result of the stimulation of the posterior SSC direction. The mean %CA values for 3,000–6,000°/s^2^ and 1,000–7,000°/s^2^ were 91.21 ± 7.96 and 91.49 ± 7.13 as a result of the stimulation of the anterior SSC direction (Table 1).

**Table 1:** Lateral, posterior, and anterior SSC %CA values.

SSC	Acceleration value	Mean ± SD	Min–Max
Lateral	1,000 °/s^2^ %CA	96.07 ± 6.61	67–100
	2,000 °/s^2^ %CA	94.58 ± 8.87	65–100
	3,000 °/s^2^ %CA	94.93 ± 9.52	55–100
	4,000 °/s^2^ %CA	93.24 ± 9.48	58.5–100
	5,000 °/s^2^ %CA	89.21 ± 11.04	50–100
	6,000 °/s^2^ %CA	83.1 ± 14.04	41.5–100
	7,000 °/s^2^ %CA	78.27 ± 18.87	33–100
	Mean %CA (4,000–6,000°/s^2^)	88.52 ± 9.04	62–100
	Mean %CA (1,000–7,000°/s^2^)	89.91 ± 6.95	68.86–100
Posterior	1,000 °/s^2^ %CA	95.26 ± 5.82	77.5–100
	2,000 °/s^2^ %CA	95.7 ± 6.5	73.5–100
	3,000 °/s^2^ %CA	94.78 ± 9.02	50–100
	4,000 °/s^2^ %CA	92.4 ± 10.62	57.5–100
	5,000 °/s^2^ %CA	89.27 ± 11.87	50–100
	6,000 °/s^2^ %CA	86.06 ± 15.08	41.5–100
	7,000 °/s^2^ %CA	84.68 ± 16.84	33–100
	Mean %CA (3,000–6,000°/s^2^)	90.63 ± 8.69	62.63–100
	Mean %CA (1,000–7,000°/s^2^)	91.16 ± 7.42	66.79–100
Anterior	1,000 °/s^2^ %CA	96.58 ± 7.55	62.5–100
	2,000 °/s^2^ %CA	95.27 ± 8.52	58.5–100
	3,000 °/s^2^ %CA	95.82 ± 6.45	76.5–100
	4,000 °/s^2^ %CA	93.3 ± 10.62	41.5–100
	5,000 °/s^2^ %CA	90.22 ± 11.6	54–100
	6,000 °/s^2^ %CA	85.53 ± 15.61	38–100
	7,000 °/s^2^ %CA	83.78 ± 15.65	41.5–100
	Mean %CA (3,000–6,000°/ s^2^)	91.21 ± 7.96	58.38–100
	Mean %CA (1,000–7,000°/s^2^)	91.49 ± 7.13	61.93–100

SD, standard deviation; Min, lowest score; max, highest score.

In the fHIT test applied to the participants included in the study, no statistically significant difference was found between the right and left ear in all acceleration values in the lateral, posterior, and anterior SSCs (*p* > 0.05) (Table 2).

**Table 2:** Comparison of lateral, posterior, and anterior SSC %CA values in the right and left ear.

SSC	Measurement	Left		Right		Test value	*p-*value
		Mean ± SD	Min–Max	Mean ± SD	Min–Max		
Lateral	1,000 °/s^2^ %CA	96.08 ± 8.03	67–100	96.05 ± 7.28	67–100	4844.5	0.614
	2,000 °/s^2^ %CA	94.15 ± 12.7	50–100	95 ± 9.54	67–100	4947.5	0.864
	3,000 °/s^2^ %CA	95.13 ± 11.5	50–100	94.73 ± 12.53	50–100	4986.5	0.960
	4,000 °/s^2^ %CA	93.75 ± 11.87	50–100	92.73 ± 13.19	50–100	4864.5	0.668
	5,000 °/s^2^ %CA	90.71 ± 13.31	50–100	87.71 ± 16	33–100	4577.5	0.248
	6,000 °/s^2^ %CA	82.36 ± 19.05	33–100	83.84 ± 18.93	33–100	4763.0	0.537
	7,000 °/s^2^ %CA	77.92 ± 22.99	20–100	78.62 ± 24.53	33–100	4895.0	0.783
	Mean %CA (4,000–6,000°/s^2^)	88.94 ± 10.9	55.67–100	88.09 ± 12.09	50–100	4950.5	0.902
	Mean %CA (1,000–7,000°/s^2^)	90.01 ± 8.12	63.29–100	89.81 ± 8.49	59.57–100	4938.5	0.880
Posterior	1,000 °/s^2^ %CA	95.53 ± 8.3	67–100	94.98 ± 9.22	67–100	4956.0	0.891
	2,000 °/s^2^ %CA	95.65 ± 9.97	67–100	95.75 ± 9.68	67–100	4991.5	0.975
	3,000 °/s^2^ %CA	95.93 ± 10.2	50–100	93.62 ± 13.27	40–100	4633.5	0.195
	4,000 °/s^2^ %CA	91.94 ± 13.13	40–100	92.86 ± 12.41	50–100	4811.5	0.569
	5,000 °/s^2^ %CA	90.28 ± 14.99	33–100	88.25 ± 14.19	50–100	4464.0	0.146
	6,000 °/s^2^ %CA	87.59 ± 20.29	25–100	84.53 ± 18.18	33–100	4363.0	0.083
	7,000 °/s^2^ %CA	84.45 ± 21.86	33–100	84.9 ± 21.22	33–100	4989.5	0.977
	Mean %CA (3,000–6,000°/s^2^)	91.44 ± 9.74	60.25–100	89.82 ± 10.62	57.5–100	4567.5	0.280
	Mean %CA (1,000–7,000°/s^2^)	91.62 ± 8.75	61.86–100	90.7 ± 9.3	59.71–100	4697.5	0.456
Anterior	1,000 °/s^2^ %CA	96.86 ± 8.82	50–100	96.29 ± 9.75	50–100	4852.5	0.567
	2,000 °/s^2^ %CA	95.19 ± 12.15	33–100	95.35 ± 10.52	60–100	4918.0	0.759
	3,000 °/s^2^ %CA	96.32 ± 8.47	67–100	95.31 ± 10.58	67–100	4975.0	0.928
	4,000 °/s^2^ %CA	93.71 ± 12.12	33–100	92.88 ± 14.65	33–100	4983.5	0.958
	5,000 °/s^2^ %CA	91.58 ± 12.89	50–100	88.85 ± 15.96	38–100	4639.0	0.312
	6,000 °/s^2^ %CA	87.15 ± 17.78	33–100	83.9 ± 20.94	25–100	4652.0	0.345
	7,000 °/s^2^ %CA	84.65 ± 20.92	33–100	82.9 ± 20.11	33–100	4697.0	0.413
	Mean %CA (3,000–6,000°/ s^2^)	92.19 ± 8.07	66–100	90.24 ± 10.81	50.75–100	4619.0	0.344
	Mean %CA (1,000–7,000°/ s^2^)	92.21 ± 7.96	66.29–100	90.78 ± 8.9	57.57–100	4540.0	0.258

SD, standard deviation; Min, lowest score; max, highest score; test value, Mann Whitney test value; *p*-value, statistical significance; *p < 0.05, there is a statistically significant difference between the groups.

In the fHIT applied to the participants included in the study, no statistically significant difference was found between the genders in the %CA values as a result of the stimulations performed at 1,000, 2,000, 3,000, 4,000, and 5,000 °/s^2^ accelerations in the lateral SSC direction (*p* > 0.05), while a statistically significant difference was found between genders in 6,000 and 7,000 °/s^2^ mean %CA values (*p* < 0.05) (Table 3).

**Table 3:** Comparison of lateral SSC %CA values according to gender.

Measurements		Sex	Mean ± SD	M (Min–Max)	Test	*p-*value
Lateral	1,000 °/s^2^ %CA	Female	95.88 ± 7.27	100(67–100)	1193.500	0.843
		Male	96.32 ± 5.66	100(75–100)		
	2,000 °/s^2^ %CA	Female	94.55 ± 9.1	100(65–100)	1206.500	0.926
		Male	94.61 ± 8.66	100(67–100)		
	3,000 °/s^2^ %CA	Female	95.03 ± 8.82	100(55–100)	1176.000	0.714
		Male	94.79 ± 10.51	100(58.5–100)		
	4,000 °/s^2^ %CA	Female	94.55 ± 8.28	100(69–100)	1035.500	0.158
		Male	91.43 ± 10.78	95.75(58.5–100)		
	5,000 °/s^2^ %CA	Female	90.87 ± 9.99	92.25(63.5–100)	1004.500	0.125
		Male	86.92 ± 12.09	87.5(50–100)		
	6,000 °/s^2^ %CA	Female	85.7 ± 14.81	87.5(41.5–100)	***866.500***	0.013*
		Male	79.51 ± 12.18	83.5(50–100)		
	7,000 °/s^2^ %CA	Female	82.1 ± 19.3	83.5(33–100)	***841.000***	0.007*
		Male	72.98 ± 17.11	75(33–100)		
	Mean %CA (4,000–6,000°/s^2^)	Female	90.37 ± 8.64	92.75(63.67–100)	***811.500***	0.004*
		Male	85.95 ± 9.06	87.33(62–100)		
	Mean %CA (1,000–7,000°/s^2^)	Female	92.24 ± 7.17	92.64(70–100)	***7888.000***	0.003*
		Male	90.21 ± 8.12	91.57(61.93–100)		

SD, standard deviation; Min, lowest score; max, highest score; test value, Mann Whitney test value; *p*-value, statistical significance; *p < 0.05, there is a statistically significant difference between the groups.

In the fHIT applied to the participants included in the study, no statistically significant difference was found between the genders in the %CA values as a result of stimulations performed at all accelerations in the posterior SSC direction (*p* > 0.05) (Table 4).

**Table 4:** Comparison of posterior SSC %CA values according to gender.

Measurements		Groups	Mean ± SD	M (Min–Max)	Test	*p*-value
Posterior	1,000 °/s^2^ %CA	Female	95.28 ± 6.13	100(78.5–100)	1170.500	0.721
		Male	95.23 ± 5.43	95(77.5–100)		
	2,000 °/s^2^ %CA	Female	95.89 ± 6.66	100(83.5–100)	1128.500	0.462
		Male	95.44 ± 6.34	100(73.5–100)		
	3,000 °/s^2^ %CA	Female	93.91 ± 10.63	100(50–100)	1168.000	0.684
		Male	95.96 ± 6.05	100(81.5–100)		
	4,000 °/s^2^ %CA	Female	93.31 ± 10.3	100(57.5–100)	1057.500	0.217
		Male	91.14 ± 11.06	94(58.5–100)		
	5,000 °/s^2^ %CA	Female	88.81 ± 12.86	91.5(50–100)	1196.000	0.875
		Male	89.89 ± 10.46	93(66.5–100)		
	6,000 °/s^2^ %CA	Female	85.78 ± 15.63	87.5(41.5–100)	1203.500	0.917
		Male	86.44 ± 14.46	87.5(50–100)		
	7,000 °/s^2^ %CA	Female	84.4 ± 17.62	90(33–100)	1209.000	0.948
		Male	85.06 ± 15.9	87.5(41.5–100)		
	Mean %CA (3,000–6,000°/s^2^)	Female	90.45 ± 9.3	92.56(62.63–100)	1200.500	0.902
		Male	90.86 ± 7.87	91.19(70.5–100)		
	Mean %CA (1,000–7,000°/s^2^)	Female	91.05 ± 8.01	93.28(66.79–100)	1179.500	0.788
		Male	91.31 ± 6.29	92.14(73–100)		

SD, standard deviation; Min, lowest score; max, highest score; test value, Mann Whitney test value; *p*-value, statistical significance; *p < 0.05, there is a statistically significant difference between the groups.

In the fHIT applied to the participants included in the study, there was no statistically significant difference between the genders in the %CA values as a result of the stimulations performed at all accelerations in the anterior SSC direction (*p* > 0.05), while a statistically significant difference was found between the genders in the mean %CA values of 3,000–6,000°/s^2^ (*p* < 0.05) (Table 5).

**Table 5:** Comparison of anterior SSC %CA values according to gender.

Measurements		Groups	Mean ± SD	M (Min–Max)	Test	*p*-value
Anterior	1,000 °/s^2^ %CA	Female	96.67 ± 7.44	100(67–100)	1161.500	0.604
		Male	96.44 ± 7.79	100(62.5–100)		
	2,000 °/s^2^ %CA	Female	95.63 ± 9.02	100(58.5–100)	1102.500	0.314
		Male	94.77 ± 7.86	100(71–100)		
	3,000 °/s^2^ %CA	Female	95.93 ± 6.52	100(76.5–100)	1184.500	0.781
		Male	95.65 ± 6.44	100(79.5–100)		
	4,000 °/s^2^ %CA	Female	95.41 ± 7.11	100(73.5–100)	979.500	0.065
		Male	90.38 ± 13.68	96.5(41.5–100)		
	5,000 °/s^2^ %CA	Female	92.63 ± 8.78	93.5(67–100)	973.000	0.073
		Male	86.88 ± 14.07	91.5(54–100)		
	6,000 °/s^2^ %CA	Female	87.09 ± 14.01	90(41.5–100)	1091.000	0.363
		Male	83.37 ± 17.54	87.5(38–100)		
	7,000 °/s^2^ %CA	Female	83.66 ± 16.12	85.5(41.5–100)	1197.500	0.883
		Male	83.94 ± 15.17	83.5(41.5–100)		
	Mean %CA (3,000–6,000°/ s^2^)	Female	92.76 ± 6.79	93.38(65.13–100)	***898.000***	0.025*
		Male	89.07 ± 9	90.63(58.38–100)		
	Mean %CA (1,000–7,000°/s^2^)	Female	92.43 ± 6.23	93.5(71.71–100)	1027.000	0.182
		Male	90.21 ± 8.13	91.57(61.93–100)		

SD, standard deviation; Min, lowest score; max, highest score; test value, Mann Whitney test value; *p*-value, statistical significance; *p < 0.05, there is a statistically significant difference between the groups.

## Discussion

VOR is a reflex that plays an important role in maintaining balance by keeping the visual field stable during head movement. Introduced in 1988 by Halmagy and Curthoys, the HIT is a test method used in VOR evaluation. With the development of the technology, the vHIT, which originated from the HIT test, was introduced in clinics where VOR evaluation is performed with video cameras. In addition to the vHIT test, which evaluates the VOR in the 5–6 Hz frequency range, the caloric test evaluating the VOR at 0.003 Hz, the rotational test evaluating the VOR at 0.05 Hz, and the head shake test evaluating the VOR at 2 Hz are different test batteries used in clinics. Pathology in the VOR arc can be determined with current vestibular tests. However, we cannot obtain complete information about the functional role of this reflex in the frequency range of our movements during daily life activities. Even if an individual’s VOR gains are normal, there may be functional impairment (12). Therefore, evaluating the gain of the VOR alone is not sufficient. The purpose of functional evaluation of the VOR is to examine visual acuity during head movements (18). Evaluation of visual acuity cannot be done with the vHIT test. The fHIT is a newly developed test battery that evaluates the functionality of the angular VOR at high acceleration/speed. Since it is a newly developed technology, there is no study in the literature in which its normative values have been determined in a healthy young population. In addition, in the use and development of the fHIT, stimulations at accelerations between 4,000 and 6,000 °/s^2^ for lateral VOR and 3,000–6,000 °/s^2^ for vertical VOR were used, whereas in our study, normative data were created by using all acceleration values between 1,000 and 7,000 °/s^2^, which is the only study in the literature covering this range in this population.

In the fHIT test, the upper limit of the %CA value is 100% and the lower limit is 0%. The closer the %CA value is to 100%, the better the functionality of the VOR. In healthy individuals, we expect the %CA values to be close to 100% as a result of the stimulation in the range of 1,000–7,000 °/s^2^ acceleration in the direction of all semicircular canals. Emekci and Erbek (13) examined the age-related changes of %CA values in the fHIT test and divided the participants into three groups: 18–35 years, 36–54 years, and 55–70 years. In the age group of 18–35 years, which overlaps the age range of our study group, the mean %CA value of the lateral SSC was 88.46, the mean %CA value of the posterior SSC was 90.54, and the mean %CA value of the anterior SSC was 88.76. In our study, the mean %CA values of lateral SSC (88.52) and posterior SSC (90.63) were similar to the study of Emekci and Erbek (13), while the mean %CA value of anterior SSC (91.21) was higher. In the study by Konukseven et al. (10) in which migraine patients without vertigo symptoms were evaluated with the fHIT, the %CA values of the control group in the lateral SSC (100–84.45), posterior SSC (100–82.60), and anterior SSC (100–85.30) were similar to the %CA values in our study. In our study, the mean %CA value was 88.94 for left lateral SSC, 88.09 for the right lateral SSC, 91.44 for the left posterior SSC, 89.82 for the right posterior SSC, 92.19 for the left anterior SSC, and 90.24 for the right anterior SSC. In a recent case–control study by Karababa et al. (19) in which individuals with motion sickness were evaluated with the fHIT, the mean %CA values of the control group for the left lateral SSC (94.37), right lateral SSC (93.75), left posterior SSC (95.71), right posterior SSC (97.57), left anterior SSC (98.33), and right anterior SSC (97.9) were higher than the values in our study. The number of individuals in the age group similar to our study by Emekci and Erbek (13) was 36 and the number of individuals in the control group in the study by Karababa et al. (19) was 35, and we think that these differences may be due to the larger population (100 individuals) in our study. In addition, Emekci and Erbek (13) and Karababa et al. (19) calculated the lateral canals values at 4,000–6,000 °/s^2^ %CA and the vertical canals 3,000–6,000 °/s^2^ %CA, whereas in our study, the lateral and vertical canal values were calculated at 1,000–7,000 °/s^2^ %CA, which is a larger range of acceleration. Therefore, %CA values may differ from the studies in the literature. In addition, it should be kept in mind that the fHIT is a perceptual test and depends on attention, memory, and motor coordination skills. In our study, in addition to the mean %CA values, %CA values at acceleration values of 1,000, 2,000, 3,000, 4,000, 5,000, 6,000 and 7,000 °/s^2^ were also analyzed separately. It was observed that %CA values decreased as the head movement acceleration increased. This is similar to the studies in the literature (10, 13, 20). As the acceleration of head movement increases, deterioration in gaze stabilization occurs (21). High velocity head movements can cause a concussion of the skull, increasing the person’s blurred vision. Therefore, even with a properly functioning VOR system, a person may be affected by this shaking and may not be able to perceive the optotype image properly. This explains the decrease in %CA values with increasing acceleration of head movement. It should be noted that the average acceleration of head movements during daily life activities is around 4,000°/s^2^. As it exceeds the limit to which the person is accustomed, there may be a decrease in visual acuity.

In our study, the mean %CA values as a result of the stimulation in the lateral SSC direction were lower than the mean %CA values obtained as a result of the stimulation in the vertical SSC direction. The same situation has been reported in the literature (13, 19). The eye moves at a shorter distance in the vertical plane and at a longer distance in the lateral plane. Visual distortions occur as the eye moves away from the center. In our study, we think that the reason why the mean %CA value of the lateral SSC was lower than the mean %CA values of the anterior and posterior SSC was due to this anatomical structure of the eye.

There are symmetrical vestibular structures on both sides of our head. In order to maintain balance, information from the right and left vestibular structures must be processed in an integrated manner. As a result of head movement, the ipsilateral vestibular structure is excited while the contralateral one is inhibited. Information from both sides must be symmetrical for the person to maintain balance (16). In our study, we examined the %CA values at different accelerations over the lateral, posterior, and anterior SSCs by differentiating between the right and left ear. We did not observe a significant difference between the right and left ear in %CA values as a result of stimulation in the direction of all SSCs at all acceleration values. In a case–control study by Teggi et al. (9) in which they conducted the fHIT in the presence and absence of optokinetic stimuli in patients with persistent postural perceptual dizziness, no statistically significant difference was observed in %CA values between the right and left ear in the control group, similar to the fHIT test performed without optokinetic stimuli. Similarly, in the case–control study of Casani et al. (22) in which they made evaluations with the fHIT in patients with vestibular migraine and acute unilateral vestibulopathy, no statistically significant difference was observed in %CA values between the right and left ear in the control group. Our study is similar to these studies in the literature. Our study shows that vestibular inputs in the right and left ear are symmetrical in healthy individuals. Therefore, no statistically significant difference was found between the right and left ear in our findings.

There is a relationship between the endocrine system and the vestibular system. Female hormones are known to trigger some vestibular system diseases (23). At the same time, although the same test procedures are applied, the test results are affected by the differences in the physical structure of men and women such as skull width and muscle mass. In our study, we examined the %CA values in the lateral, posterior, and anterior SSCs at different accelerations according to gender. We observed statistically significant differences between genders in the lateral SSC 6,000, 7,000 °/s^2^ mean %CA values and in the mean %CA values of 3,000–6,000 °/s^2^ in the anterior SSC. On the contrary, in the study of Emekci and Erbek (13), no significant difference was observed between genders at different acceleration values in all SSCs. In different studies in the literature, no significant difference was observed between genders in gaze stabilization test (24) and VOR gain values (25, 26). The findings in our study differ from the literature in this regard. We think that these differences may be due to hormonal, anatomical, and physical differences.

## Conclusion

Our study reveals the importance of frequency-based functional evaluation of the VOR at different accelerations in young adults. Determination of normative values at all accelerations (1,000–7,000 °/s^2^) in clinical use constitutes an important database for future studies to distinguish pathological results. We think that our study data can be a guide for the vestibular rehabilitation process by evaluating different frequencies of the VOR arc in patients with balance problems. By preparing exercise programs according to the affected VOR frequency, it will enable the targeted treatment process to begin. It can also be used for the prognosis of the disease and the follow-up of the rehabilitation process.

## Limitations

The limitations of the present study, in which we aimed to determine the normative values of the fHIT test in healthy young adults, include the fact that different experts administered the test, the sample size, and the lack of objective tests to distinguish auditory and vestibular pathologies in the exclusion criteria of the study.

## Data availability statement

The raw data supporting the conclusions of this article will be made available by the authors, without undue reservation.

## Ethics statement

Approval was obtained from the İnönü University's Institute of Health Sciences Non-Interventional Clinical Research Ethics Committee (decision number: 2022/3465) and all individuals participating in the study. The studies were conducted in accordance with the local legislation and institutional requirements. The participants provided their written informed consent to participate in this study.

## Author contributions

DC: Conceptualization, Methodology, Project administration, Supervision, Writing – original draft. HE: Conceptualization, Methodology, Supervision, Writing – review & editing. SÇ: Writing – original draft. BüK: Writing – original draft. SD: Writing – original draft. EK: Writing – review & editing. BuK: Writing – original draft. EÖ: Writing – original draft. MI: Writing – review & editing. İD: Writing – review & editing.
